# Sanjad-Sakati Syndrome and Its Association with Superior Mesenteric Artery Syndrome

**DOI:** 10.1155/2014/108051

**Published:** 2014-11-09

**Authors:** Osamah Abdullah AlAyed

**Affiliations:** King Faisal Specialist Hospital Research Centre, P.O. Box 280581, Riyadh 11392, Saudi Arabia

## Abstract

Sanjad-Sakati syndrome (SSS) is an autosomal recessive disorder found exclusively in people of Arabian origin. It was first reported in the Kingdom of Saudi Arabia in 1988 and confirmed by a definitive report in 1991. The syndrome comprises of congenital hypoparathyroidism, seizures, severe growth and developmental retardation, low IQ, and atypical facial features. Supportive treatment in the form of vitamin D and growth hormone supplementation is often offered to patients suffering from SSS. This case study focuses on the steps taken to help a patient who was found to have very unusual symptoms and was later found to have superior mesenteric artery syndrome.

## 1. Introduction

Sanjad-Sakati syndrome (SSS) is a newly described syndrome found mainly in the Middle East and Arabian Gulf countries. The condition was first reported by Sanjad et al. in 1988 [1]. Three years later, its inheritance and configuration were confirmed by the same team from King Faisal Specialist Hospital and Research Centre, Saudi Arabia [2]. Later on, the syndrome was also described by Richardson and Kirk in 1990 [3] and in 1992 by Kalam and Hafeez [4]. SSS is listed in OMIM (241410) as HRD, that is, hypoparathyroidism-retardation-dysmorphism Syndrome. Parvari et al. reported a gene location on chromosome number 1 at 1q42-43, this syndrome can be caused by mutations in the gene encoding tubulin-specific chaperone E (*TBCE*) [5]. Children affected with this condition develop poorly in the mother's womb (IUGR) and therefore, after birth, suffer from various conditions such as hypocalcemic tetany or seizures due to hypoparathyroidism at an early stage in their lives [2]. They have atypical physical features, namely, long narrow faces, deep set small eyes, beaked noses, and large ears that have tendency to droop and undersized jaws (micrognathia). Along with the physical abnormalities, children suffering from SSS also exhibit mild to moderate mental retardation, leading to poor life prospects.

Less superficial symptoms have also been documented, namely, skeletal defects (medullary stenosis), hypocalcaemia, hyperphosphatemia, and low concentration of immunoreactive parathyroid hormone. In some rare cases, there may even be complete respiratory failure eventually [6, 7].

## 2. Case Report

An 8-year-old girl was diagnosed with Sanjad-Sakati syndrome (SSS) and was prescribed calcium and vitamin D supplements (one-alpha drops). She was in her usual state of health until January 2013 when she suddenly began to suffer from frequent vomiting with no apparent reason. She immediately went to her local hospital where she was diagnosed with gastroenteritis and treated accordingly. However, the problem persisted for a time and then worsened, adding severe constipation (only passing stool every three days) to the list of symptoms. She was once again admitted to the hospital and, once again, treated for the same condition. As her problem did not improve, she went to her local clinic and demanded the opinion of an expert. A gastroenterology team was consulted and, following their advice, she undertook a barium follow through. The results showed all the symptoms of severe superior mesenteric artery syndrome, a condition that causes almost complete intestinal obstruction at the level of the third part of the duodenum. With this new information, she was readmitted to the hospital on January 11 and underwent an upper esophagogastroduodenoscopy and NJ tube insertion. A biopsy was also taken because of the severe difficulties in performing the endoscopy. Despite all this, her condition worsened and, on January 15, she suffered an episode of vomiting, choking, and aspiration pneumonia, which required an ICU admission of 10 days for a course of antibiotics. After this, she was allowed to start gradual NJ feeding with a small amount administered initially (2 cc) and an incremental increase until the full dose (45 cc) was reached. She tolerated this well and it was deemed safe to prescribe her Movicol for the constipation. The medication worked and her bowel obstruction began to clear in the form of diarrhea 3 times a day, which required various readjustments of the NJ tube and even the insertion of an NG tube for drainage.

## 3. Discussion

Sanjad-Sakati syndrome tends to have severe growth retardation which is usually the predisposing factor in developing superior mesenteric artery (SMA) syndrome.

SMA syndrome is an uncommon but well-recognized clinical entity characterized by compression of the third, or transverse, portion of the duodenum between the aorta and the superior mesenteric artery. This results in chronic, intermittent, acute, complete or partial duodenal obstruction [8]. Superior mesenteric artery syndrome was first described in 1861 by Von Rokitansky, who proposed that its cause was obstruction of the third part of the duodenum as a result of arteriomesenteric compression. Some studies report the incidence of superior mesenteric artery syndrome to be 0.1–0.3% [9].

But the diagnosis of SMA syndrome is difficult. Confirmation usually requires radiographic studies, such as an upper GI series, hypotonic duodenography, and CT scanning. Therefore, there are very few studies that report the incidence of superior mesenteric artery syndrome (0.1–0.3%) and approximately 0.013–0.78% of barium upper GI studies evaluating the condition support the diagnosis [10, 11].

What can be confirmed from this study is that the insertion of an NJ to bypass the obstruction proved very helpful, allowing the patient to tolerate food, to gain weight, and to increase the angle between the superior mesenteric artery and the third part of the duodenum, thus relieving the symptoms. Reversing or removing the precipitating factor is usually successful in a patient with acute superior mesenteric artery (SMA) syndrome.

Conservative initial treatment is recommended in all patients with superior mesenteric artery syndrome; this includes adequate nutrition, nasogastric decompression, and proper positioning of the patient after eating (i.e., left lateral decubitus, prone, knee-to-chest position, or Goldthwaite maneuver). Enteral feeding using a double lumen nasojejunal tube passed distal to the obstruction under fluoroscopic assistance is an effective adjunct in treatment of patients with rapid severe weight loss and also eliminates the need for intravenous fluids and the risks associated with total parenteral nutrition.

In some instances, both enteral and parenteral nutritional support may be needed to provide optimal calories. The patient's weight should be monitored daily. Subsequently, the patient can be started on oral liquids followed by slow and gradual introduction of small and frequent soft meals as tolerated. Finally, regular solid foods are introduced.

Metoclopramide treatment may be beneficial.

Surgical intervention is indicated when conservative measures are ineffective, particularly in patients with a long history of progressive weight loss, pronounced duodenal dilatation with stasis, and complicating peptic ulcer disease. A trial of conservative treatment should be instituted for at least 4–6 weeks prior to surgical intervention.

Options for surgery include a duodenojejunostomy or gastrojejunostomy to bypass the obstruction or a duodenal derotation procedure (otherwise known as the Strong procedure) to alter the aortomesenteric angle and place the third and fourth portions of the duodenum to the right of the superior mesenteric artery.

## 4. Conclusion

To the best of our knowledge and as far back as we go through previous case reports, this is the first case that showed that there could be a relation between SSS and SMA syndrome. It was therefore the aim of this case report to emphasize the importance of considering superior mesenteric artery syndrome when dealing with Sanjad-Sakati syndrome sufferers who present symptoms of intestinal obstruction. We hope that, by doing so, we may enable medical practitioners to better assess and treat future situations of this nature.

## References

[1] Sanjad S., Sakati N., Abu-Osba Y. (1988). Congenital hypoparathyroidism with dysmorphic features: a new syndrome. *Pediatric Research*.

[2] Sanjad S. A., Sakati N. A., Abu-Osba Y. K., Kaddoura R., Milner R. D. G. (1991). A new syndrome of congenital hypoparathyroidism, severe growth failure, and dysmorphic features. *Archives of Disease in Childhood*.

[3] Richardson R. J., Kirk J. M. W. (1990). Short stature, mental retardation, and hypoparathyroidism: a new syndrome. *Archives of Disease in Childhood*.

[4] Kalam M. A., Hafeez W. (1992). Congenital hypoparathyroidism, seizure, extreme growth failure with developmental delay and dysmorphic features—another case of this new syndrome. *Clinical Genetics*.

[5] Parvari R., Hershkovitz E., Kanis A., Gorodischer R., Shalitin S., Sheffield V. C., Carmi R. (1998). Homozygosity and linkage-disequilibrium mapping of the syndrome of congenital hypoparathyroidism, growth and mental retardation, and dysmorphism to a 1-cM interval on chromosome 1q42-43. *The American Journal of Human Genetics*.

[6] Parvari R., Hershkovitz E., Grossman N., Gorodischer R., Loeys B., Zecic A., Mortier G., Gregory S., Sharony R., Kambouris M., Sakati N., Meyer B. F., Al Aqeel A. I., Al Humaidan A. K., Al Zanhrani F. A., Al Swaid A., Al Othman J., Diaz G. A., Weiner R., Khan K. T. S., Gordon R., Gelb B. D. (2002). Mutation of TBCE causes hypoparathyroidism-retardation-dysmorphism and autosomal recessive Kenny-Caffey Syndrome. *Nature Genetics*.

[7] Parvari R., Hershkovitz E., Grossman N., Gorodischer R., Loeys B., Zecic A., Mortier G., Gregory S., Sharony R., Kambouris M., Sakati N., Meyer B. F., Al Aqeel A. I., Al Humaidan A. K., Al Zanhrani F. A., Al Swaid A., Al Othman J., Diaz G. A., Weiner R., Khan K. T. S., Gordon R., Gelb B. D. (2002). Mutation of TBCE causes hypoparathyroidism-retardation-dysmorphism and autosomal recessive Kenny-Caffey Syndrome. *Nature Genetics*.

[8] Gerasimidis T., George F. (2009). Superior mesenteric artery syndrome. *Digestive Surgery*.

[9] Shiu J.-R., Chao H.-C., Luo C.-C., Lai M.-W., Kong M.-S., Chen S.-Y., Chen C.-C., Wang C.-J. (2010). Clinical and nutritional outcomes in children with idiopathic superior mesenteric artery syndrome. *Journal of Pediatric Gastroenterology and Nutrition*.

[10] Baltazar U., Dunn J., Floresguerra C., Schmidt L., Browder W. (2000). Superior mesenteric artery syndrome: an uncommon cause of intestinal obstruction. *Southern Medical Journal*.

[11] Ünal B., Aktaş A., Kemal G., Bilgili Y., Güliter S., Daphan Ç., Aydinuraz K. (2005). Superior mesenteric artery syndrome: CT and ultrasonography findings. *Diagnostic and Interventional Radiology*.

